# Sources of seasonal wetland methane emissions in permafrost regions of the Qinghai-Tibet Plateau

**DOI:** 10.1038/s41598-020-63054-z

**Published:** 2020-05-05

**Authors:** Shunyao Zhang, Fugui Zhang, Zeming Shi, Aihua Qin, Huiyan Wang, Zhongjun Sun, Zhibin Yang, Youhai Zhu, Shouji Pang, Pingkang Wang

**Affiliations:** 1 College of Earth Sciences, Chengdu University of Technology, Chengdu, 610059 China; 2 Institute of Geophysical & Geochemical Exploration, Chinese Academy of Geological Sciences, Langfang, 065000 China; 3 Key Laboratory of Geochemical Cycling of Carbon and Mercury in the Earth’s Critical Zone, Chinese Academy of Geological Sciences, Langfang, 065000 China; 4 Oil and Gas Survey, China Geological Survey, Beijing, 100029 China; 5 Muli Field Scientific Observation and Research Station for Gas Hydrate, China Geological Survey, Beijing, 100029 China

**Keywords:** Projection and prediction, Geochemistry

## Abstract

In this study, systematic soil methane cycle geochemical monitoring was carried out in a typical gas hydrate region in the Qinghai-Tibet Plateau. Soil gas samples were collected for hydrocarbon components and carbon isotope analysis. Meanwhile, soil-methane fluxes from the upper active layer (20–30 cm) were monitored during six months of one year. The results of this research provide evidence of a new source of methane emission from wetland soils in permafrost regions: gas hydrate release. Sites with large methane emissions were found using flux monitoring, the characteristics of thermogenic methane were identified using carbon isotope tracing, and the relationship between emission by soils and effusion from gas hydrates was determined through correlation analyses of soil-adsorbed hydrocarbons. Seasonal variation of methane emissions are also discussed by considering the emission of bacterial methane, thermogenic methane, and the absorption of methane from the soil active layer. These comprehensive findings provide valuable information for carbon cycle research of wetlands in permafrost regions.

## Introduction

Methane is the second most abundant greenhouse gas in the atmosphere^1,2^. Since 2007, global atmospheric methane concentrations have increased rapidly at an annual rate of more than 6.8‒10 ppb^3,4^, and the consequent increase in radiative forcing is one of the reasons for a continued rise in global temperatures^5^. Studies have shown that climate warming will lead to the release of carbon from soil pools^6^, resulting in a positive feedback effect^7^. The emission of methane plays a pivotal role in such positive land-based carbon climate feedback mechanisms^8,9^. As one of the largest carbon pools in nature, wetlands in permafrost regions release 20‒72 Tg of methane every year^10,11^. This release is modulated by methane sources and varies across the seasons. Therefore, studies on the seasonal variations of methane emission sources are of great significance to understanding wetland methane emission processes, investigating wetland carbon cycling, and evaluating regional greenhouse effects.

The Qinghai-Tibet Plateau contains the largest distribution of frozen alpine soil in the world, with a wetland area spanning approximately 130,000 km^2^. Being one of the key ecological safety barriers in Asia^12^, the rate of increase of the atmospheric background methane concentration in the Qinghai-Tibet Plateau is significantly higher than the global average^13^. Recent research has found that global wetland methane emissions increase continuously during the process of climate warming^4^. There are two main theories that explain this phenomenon: (1) elevated temperatures promote soil microbial activity at deeper horizons, thus increasing the environment’s carrying capacity for methanogenic microbial communities and increasing methane emissions from microbes^14^; (2) plant activity increases with temperature, thereby influencing the methanogenic microbial community structures and promoting bacterial methane emissions^15^. Compared with conventional peat wetlands, the wetlands in the permafrost regions of the Qinghai-Tibet Plateau possess high-altitude alpine climatic characteristics, which result in higher sensitivities of the wetland ecosystems to temperature changes. In addition, substantial amounts of soil organic carbon^16^ and thermogenic hydrocarbons in gas hydrates^17^ are stored in the Qinghai-Tibet Plateau. These hydrocarbons contribute to methane emission mechanisms that are unique to the region.

Studies on carbon cycling in the wetlands of the Qinghai-Tibet Plateau indicate that elevated temperatures can increase plant and microbial activity, which can lead to increased soil organic carbon content^18^ and increased wetland methane emissions^19^. In particular, methane emission bursts have been observed in wetlands during the growing season^20–22^. Mackelprang *et al*.^23^ attributed the methane emission bursts in wetlands of permafrost regions to methane releases from deep permafrost layers. Lu *et al*.^24^ asserted that the source of methane emissions from wetland surfaces in the Qinghai-Tibet Plateau could be hydrocarbon gases released by subterranean gas hydrates^24–26^. Sun *et al*.^17^ pointed out that hydrocarbon gases in gas hydrates can effuse towards the land surface through microseepage. According to the hydrocarbon microleakage theory, the hydrocarbon gases in the effusion of gas hydrates may have an impact on the surface carbon circulation system, forming a key source of wetland methane emissions. However, there was lack of evidence for the contribution of the hydrocarbon in gas hydrates to soil methane emissions. Therefore, in studies on wetland methane emissions in the permafrost regions of the Qinghai-Tibet Plateau, the effusion of methane from gas hydrates—which has not been previously explored in detail—must also be considered.

The gas hydrates of the permafrost regions of the Qinghai-Tibet Plateau were first observed in 2008. They are type II hydrates that mainly occur in the pores and fissures of fine-grained sandstones, siltstones, mudstones, and oil shales of the Middle Jurassic Jiangcang Formation, at depths of 133‒396 m^24,27,28^. The hydrocarbons in gas hydrates are mainly thermogenic, with a dryness coefficient (C_1_/C_2+3_) ranging from 1.3 to 26.0 and a δ^13^C methane value ranging from −52.7‰ to −35.8‰^29^. As the δ^13^C content of methane between thermogenic and bacterial gases differs, researchers proposed diagrams to distinguish between methane gas sources^30–35^. However, few studies on methods to distinguish between the sources of wetland methane gas in the Qinghai-Tibet Plateau have been reported.

**Figure 1:**
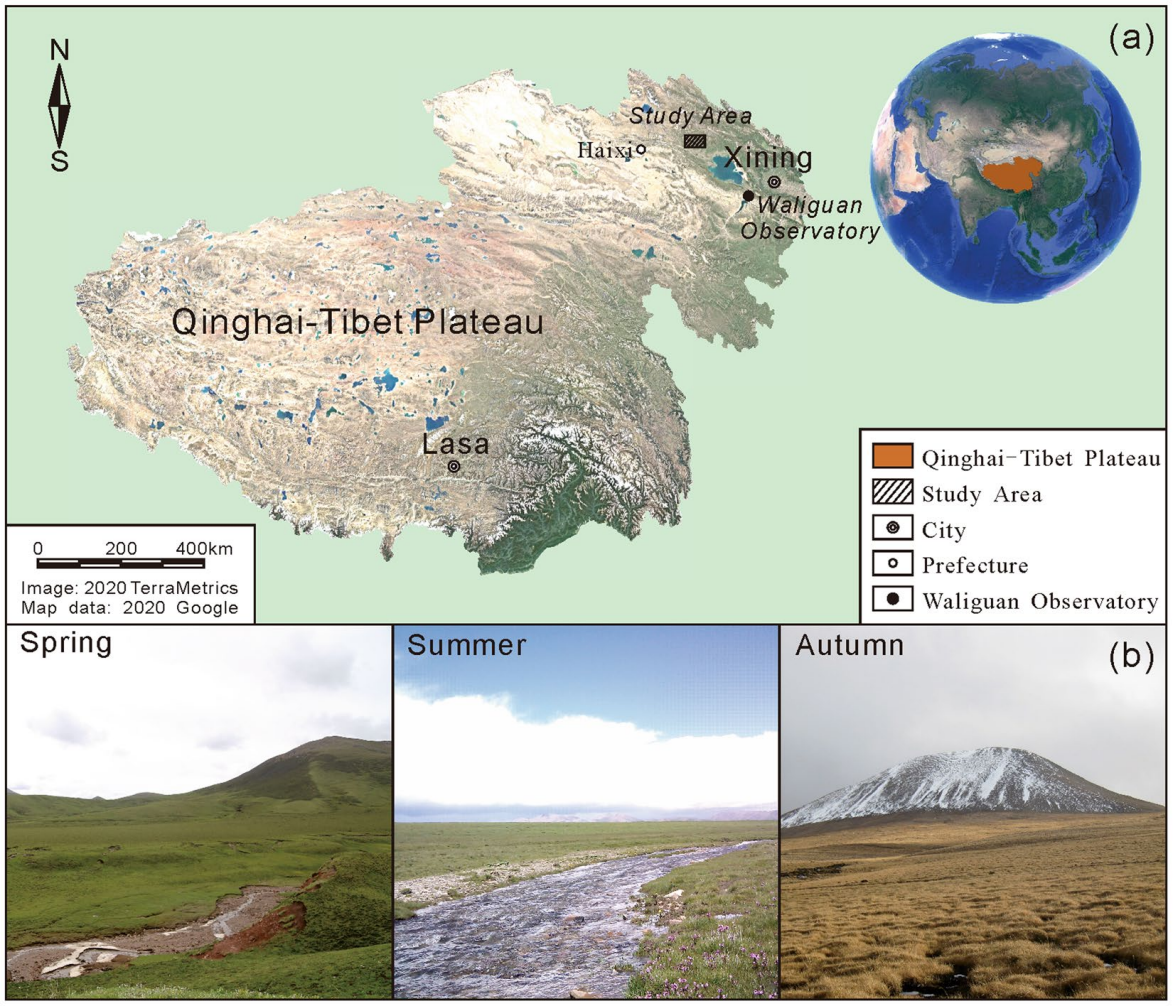
(**a**) Location of study area, and (**b**) landscapes during various seasons. Remote sensing image of the Qinghai-Tibet plateau generated using Google Earth Pro 7.1.8.3036 (Image: 2020 TerraMetrics; Map data: 2020 Google), URL: https://www.google.com/earth/, the final figure was generated using CorelDRAW X6, URL: https://www.coreldraw.com/en/product/coreldraw/. Photos taken by Shunyao Zhang at Muli Field Scientific Observation and Research Station, Haixi, Qinghai.

In order to get an understanding of the source and seasonal variation patterns of methane emissions, in this study, a systematic soil methane cycle geochemical monitoring has been carried out in the Muli area located in the northeastern region of the Qinghai-Tibet Plateau (Fig. 1a). As a typical wetland, the study area is situated 4100–4300 m above sea level. The soil active layer thickness and permafrost thickness are 2.4 and 80 m, respectively^36^. The wetland ecosystem is mainly influenced by freezing and thawing of the permafrost which is governed by seasonal temperature variations (Fig. 1b). Microbes and thermogenic methane were characterized according to the δ^13^C content of methane and the seasonal variation patterns of methane emission were discussed based on the geochemical analysis. At present, the methane emissions of the wetland soil in the Qinghai-Tibet plateau have not been investigated in full, and our study provides a novel way of source apportionment for soil methane emissions in permafrost regions.

## Results

### Methane content of low-level air and soil-adsorbed gas

**Figure 2:**
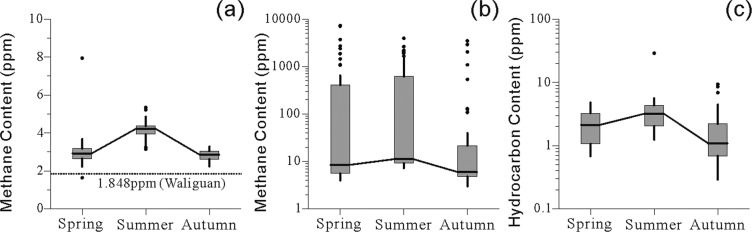
The boxplot represents seasonal variation of (**a**) methane content of low-level air, (**b**) methane content of near-surface soil-adsorbed gas, and (**c**) the heavy hydrocarbon content of soil-adsorbed gas. Median, 25th and 75th quantiles are shown in the box; data outside the 1.5 × interquartile range regarded as outliers and shown as points. The mean methane content of Waliguan cited from WMO, 2017. Heavy hydrocarbon content is the sum of ethane, ethylene, propane, propylene, butane, and pentane.

Table 1 shows the methane content of the low-level air and soil-adsorbed gas samples that were collected. The results (Fig. 2) indicate that the near-surface low-level air data had the smallest dispersion, with a coefficient of variation ranging from 9.62% to 29.00%. The methane concentration of low-level air was significantly higher than that of the regional base air (Fig. 2a). Data on the methane content of near-surface soil-adsorbed gas had the largest dispersion, with a coefficient of variation ranging from 194.37% to 294.43% (Fig. 2b). Data on the heavy hydrocarbon content of near-surface soil-adsorbed gas had a smaller dispersion, with a coefficient of variation ranging from 52.99% to 111.31% (Fig. 2c). The methane content of low-level air and soil-adsorbed gas, as well as the heavy hydrocarbon content (C_2_–C_5_) of soil-adsorbed gas exhibited consistent seasonal variation trends, with the highest values in summer, followed by spring and autumn.

**Table 1:** Methane content in low-level air and soil-adsorbed gas.

	Absorption methane content (ppm)			Absorbed heavy hydrocarbon content (ppm)			Absorption dryness coefficient (nodim)			Base air methane content (ppm)		
	Spring	Summer	Autumn	Spring	Summer	Autumn	Spring	Summer	Autumn	Spring	Summer	Autumn
No. of samples	42	42	42	42	42	42	42	42	42	42	42	42
Maximum value	7272.98	3958.93	3470.45	4.83	28.95	9.38	2477.27	1556.48	421.12	7.94	5.34	3.29
Minimum value	3.98	7.17	3.00	0.68	1.24	0.29	2.29	3.22	3.24	1.64	3.15	2.24
Mean	736.73	465.04	256.89	2.26	3.77	1.99	241.39	150.76	49.45	3.02	4.17	2.82
Median	8.49	11.22	5.97	2.13	3.21	1.09	9.74	7.67	9.26	2.92	4.22	2.85
Standard deviation	1682.34	903.90	756.36	1.20	4.19	2.09	549.64	312.49	106.27	0.88	0.45	0.27
Coefficient of variation	228.35%	194.37%	294.43%	52.99%	111.31%	105.17%	227.70%	207.27%	214.91%	29.00%	10.89%	9.62%

The correlation between methane content and heavy hydrocarbon content in surface soil-adsorbed gas can reflect the effusion of thermogenic gases that formed underground^17^. Geochemical analyses of cores from gas hydrate wells in the study area revealed that the gaseous portion of gas hydrates is dominated by methane and also contains a substantial amount of heavy hydrocarbons^27,29^. Such gases are generated by the large-scale migration of hydrocarbon gases in deep source rocks^37^. During the upward effusion of gases from gas hydrates, methane and heavy hydrocarbons simultaneously effuse towards the surface, thereby directly influencing the hydrocarbon content of near-surface soil-adsorbed gas.

Table 2 shows the results of correlation analyses performed on the collected data. The correlation coefficients between methane content and heavy hydrocarbon content in spring, summer, and autumn were 0.48, 0.17, and 0.88, respectively. Previous studies have reported that bacterial gases have a high methane content and an extremely low heavy hydrocarbon content^38^. The methane content and heavy hydrocarbon content during autumn were significantly correlated (R = 0.88), which indicates that effusion from gas hydrates was the main contributor to methane at wetland surfaces. In contrast, the effusion of underground hydrocarbons did not produce a significant influence on the land surface during spring and summer.

**Table 2:** Correlation coefficients corresponding to hydrocarbon indicators of near-surface adsorbed gas (nodim).

		Spring		Summer		Autumn	
		CH_4_	C_2_^+^	CH_4_	C_2_^+^	CH_4_	C_2_^+^
Spring	CH_4_	1.00					
	C_2_^+^	0.48	1.00				
Summer	CH_4_	0.17	0.49	1.00			
	C_2_^+^	0.67	0.34	0.17	1.00		
Autumn	CH_4_	0.29	0.30	0.38	0.00	1.00	
	C_2_^+^	0.29	0.46	0.36	0.00	0.88	1.00

### Carbon isotope content of methane

**Figure 3:**
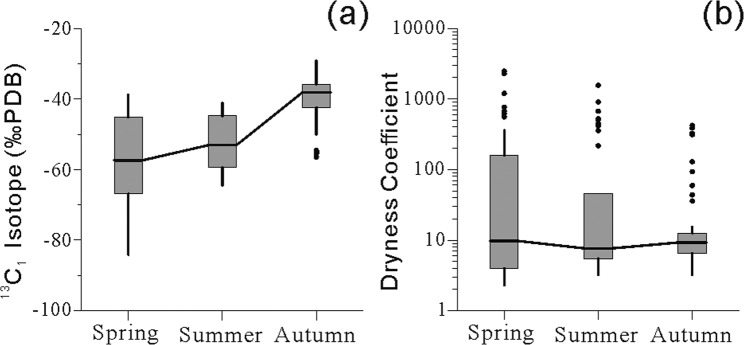
The boxplot represents seasonal variation of (**a**) the carbon isotopes of methane in free gas of the active soil layer, and (**b**) the dryness coefficient of adsorbed gas. Median, 25th and 75th quantiles are shown in the box; data outside the 1.5× interquartile range regarded as outliers and shown as points. The calculation of dryness coefficient refers to Bernard et al., 1976.

Table 3 shows the carbon isotope content of methane for the collected samples. The results (Fig. 3) indicate that samples collected in the spring had the lowest mean carbon isotope value of –57.27‰ Peedee Belemnite (PDB) formation from South Carolina, USA, as well as the largest dispersion with a coefficient of variation of –23.19%. Samples collected in the summer showed a lower methane carbon isotope content (mean value of –52.52‰ PDB) and a smaller dispersion (coefficient of variation of –14.93%). Samples collected in the autumn showed the lowest carbon isotope methane content (mean value of –39.78‰ PDB) and the smallest dispersion (coefficient of variation of –16.25%).

**Table 3:** Carbon isotopic (δ^13^C_1_) content of methane in the free gas of the soil active layer (‰PDB).

	No. of samples	Maximum value	Minimum value	Mean	Median	Standard deviation	Coefficient of variation
Spring	42	–38.76	–84.11	–57.27	–57.41	13.28	–23.19%
Summer	42	–41.16	–64.45	–52.52	–53.09	7.84	–14.93%
Autumn	42	–29.16	–56.45	–39.78	–38.16	6.46	–16.25%

**Figure 4:**
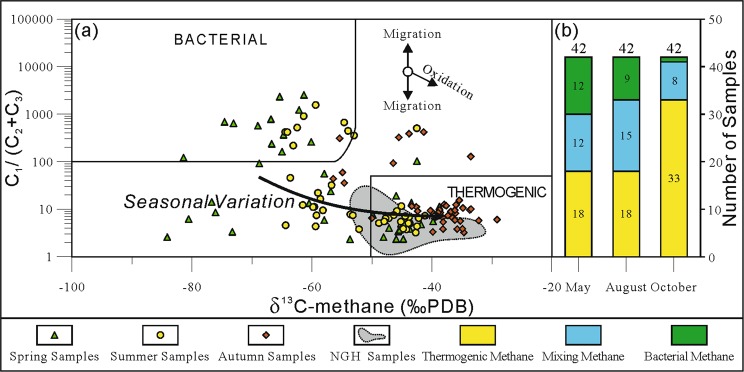
Seasonal variation of near-surface methane sources. (**a**) The interpretative diagram of methane is referenced from Bernard *et al*. (1976), Whiticar (1999). Bacterial source is the microbial produces, thermogenic source is the thermo genic generation of hydrocarbons. Data points are obtained through this experiment. (**b**) Samples numbers of different methane source. Nature gas hydrate (NGH) samples data cited from Dai *et al*., 2017.

The δ^13^C_1_ content of methane and the dryness coefficient of gas hydrates can indicate the source of hydrocarbon gas: δ^13^C_1_ < –55‰ PDB indicates bacterially-derived methane, and δ^13^C_1_ > –50‰ PDB indicates thermogenically-derived methane^31^. The Bernard diagram^30^ was used to distinguish between gas sources, the results of which are shown in Fig. 4a. The methane gas sources in the study area exhibited strong polarization during spring. Among the samples collected in spring, 18 were thermogenically-derived methane (43% of all samples) with δ^13^C_1_ contents of –49.86‰ to –38.76‰ and dryness coefficients of 2.30 to 18.68; 12 samples were bacterially-derived methane (29% of all samples), with δ^13^C_1_ contents of –81.39‰ to –60.12‰ and dryness coefficients of 119.09 to 2477.27. The methane sources in summer were similar to those in spring: 18 samples were thermogenically-derived methane (43% of all samples) with δ^13^C_1_ contents of –48.85‰ to –41.16‰ and dryness coefficients of 3.23 to 11.64; 9 samples were bacterially-derived methane (21% of all samples) with δ^13^C_1_ contents of –64.45‰ to –52.99‰ and dryness coefficients of 218.49 to 1556.48. In autumn, the surface methane exhibited a strong thermogenic nature, with 33 samples being thermogenically-derived methane (70% of all samples) with δ^13^C_1_ contents of –50.00‰ to –29.16‰ and dryness coefficients of 3.24 to 15.46; and only one sample was bacterially-derived methane (Fig. 4b).

**Figure 5:**
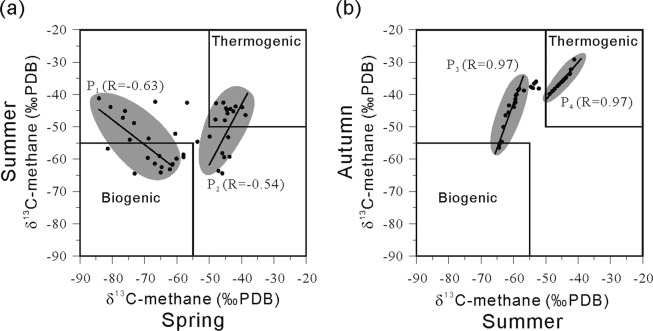
Seasonal variations of the carbon isotope content of methane in free gas of the soil active layer; (**a**) spring to summer, and (**b**) summer to autumn.

Compared with the actual gas hydrate samples, the thermogenic samples in the three groups had identical δ^13^C_1_ values and slightly higher dryness coefficients. Results of a gas hydrate simulation experiment indicated that methane exhibits the greatest migration extent during the upward effusion of underground hydrocarbon gases. In contrast, heavy hydrocarbons (C_2_–C_5_) tend to enter the hydrate lattices^39^. This fractional distillation process during hydrocarbon effusion leads to an increase in the dryness coefficient of the soil active layer-adsorbed gas. The samples from all three seasons contained outliers characterized by lighter carbon isotopes (>–55‰ PDB) and higher dryness coefficients (>100) (Fig. 5b), which are generally indicative of bacterially-derived hydrocarbons^30,31^.

### Seasonal variation of methane sources

The observed variations in the carbon content of methane reflect the seasonal variations in methane gas sources. As shown in Fig. 5a, the relationship between the carbon isotope contents of spring and summer samples can be generally divided into two parts with correlation coefficient values of –0.63 and 0.54. The P_1_ zone consists of 16 samples: the spring samples containing bacterially-derived methane with δ^13^C_1_ contents of –84.11‰ to –62.15‰ PDB, and the summer samples having heavier isotopic contents, higher proportions of thermogenically-derived methane, and δ^13^C_1_ contents of –64.45‰ to –41.16‰ PDB. In this zone, the dominant sources of wetland methane gas changed from bacterial sources to bacterial and mixed sources. The P_2_ zone consists of 16 samples: the spring samples containing thermogenically-derived methane with δ^13^C_1_ contents of –47.15‰ to –38.76‰ PDB, and the summer samples having lighter isotopic contents, increased proportions of bacterial methane, and δ^13^C_1_ contents of –64.39‰ to –42.90‰ PDB. In this zone, the dominant sources of wetland methane gas changed from thermogenic sources to bacterial sources. The characteristics of source variations in both zones indicate that changes occurred in both bacterial and thermogenic gas sources during the transition from spring to summer.

Figure 5b shows the relationship between the carbon isotope contents of summer and autumn samples can also be generally divided into two parts with correlation coefficient values of 0.97. The P_3_ zone consists of 16 samples, with the summer samples containing bacterially-derived methane with δ^13^C_1_ contents of –64.45‰ to –58.17‰ PDB. The P_4_ zone consists of 19 samples, with summer δ^13^C_1_ contents of –48.85‰ to –41.16‰ PDB and autumn δ^13^C_1_ contents of –48.85‰ to –41.16‰ PDB, which indicates heavier carbon isotope content. The consistent characteristics of source variations in both zones indicates a decrease in bacterial activity and an increase in thermogenic production during the transition from summer to autumn, resulting in wetland methane gas sources being dominated by thermogenically-derived sources during autumn.

### Methane flux from the upper active layer

**Figure 6:**
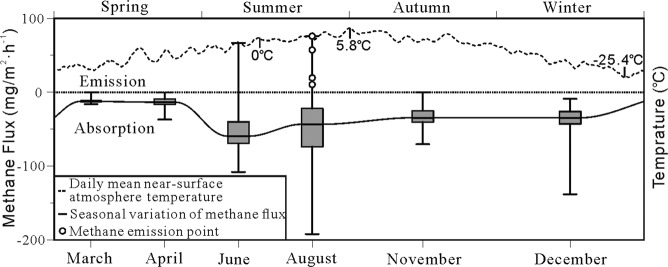
The relationship between seasonal methane flux from the soil active layer and atmospheric temperature. The boxplot represents the variance of methane flux. Median, 25th and 75th quantiles are shown in the box; whiskers indicate the minimal and maximal values.

Recent studies have suggested that the upper active layer of soil (0–30 cm) in wetlands is an important methane sink^18,40,41^. This absorption can greatly affect the soil methane emission. Table 4 shows the soil-atmosphere methane flux data of the upper active layer obtained from the experiments described in Section 2. Figure 6 shows the annual variation of methane absorption in the soil active layer. The mean monthly methane absorption by soil was –11.47 mg/m^–2^·h^–1^ in March and gradually increased until April; in June, the methane absorption flux increased rapidly, reaching a maximum value of –52.61 mg/m^–2^·h^–1^. In August, the methane absorption flux decreased gradually, with a mean value of –46.26 mg/m^–2^·h^–1^. Changes in methane absorption flux were gradual throughout November to December, with mean absorption flux values of –33.01 mg/m^–2^·h^–1^ and –36.57 mg/m^–2^·h^–1^, respectively.

**Table 4:** Methane flux in the upper active layer during various seasons (mg/m^–2^·h^–1^).

	Spring		Summer		Autumn	Winter
	March	April	June	August	November	December
No. of samples	42	42	42	42	42	42
Maximum value	0.00	–0.40	66.93	76.00	0.00	–8.93
Minimum value	–16.13	–37.07	–108.00	–192.00	–70.40	–138.13
Mean	–11.47	–13.81	–52.61	–46.26	–33.01	–36.57
Median	–12.40	–13.47	–59.40	–43.27	–34.47	–34.47
Standard deviation	3.65	7.46	33.99	48.31	13.84	20.52
Coefficient of variation	–31.81%	–54.00%	–64.60%	–104.43%	–41.92%	–56.12%

The methane absorption effect of soil was relatively weak in spring, strengthened during early summer, gradually weakened towards the end of summer, and became weak again in autumn and winter. From March to April, both the atmospheric temperature and methane absorption flux increased gradually; from April to June, the atmospheric temperature increased further, prompting the onset of the growing season for wetland organisms and causing a rapid increase in methane absorption flux, consistent with results obtained by Turestsky and Vitt^42^. From June to August, as the atmospheric temperature increased above 0 °C, the permafrost thaw depth peaked and the methane absorption flux gradually decreased. Studies have shown that permafrost thaw leads to changes in soil temperature and wetland water levels^43,44^, which promotes methane emissions by microbes under anaerobic conditions^45,46^. Elevated temperatures also increase the activity of methanotrophic microbes in plants and soil at wetland surfaces, resulting in an increase in methane absorption flux and maintenance of the carbon sink function of the upper active layer^18^. From June to August, the methane absorption flux in the upper active layer exhibited a decreasing trend, which indicates that the increase in wetland methane emissions exceeded the increase in methane absorption by the soil, leading to changes in the carbon source–carbon sink dynamic geochemical balance in wetland soil.

Previous studies suggested that the gas hydrate mining activities did not affect surface greenhouse gas composition^47^. However, in August, four high methane emission fluxes were observed, with values of 10.40–76.00 mg/m^–2^·h^–1^. The carbon isotope (δ^13^C_1_) content of methane in the free gas at the methane emission sites was between –47.96‰ and –42.22‰ PDB, which corresponds to thermogenic methane. Therefore, the decrease in the methane absorption effect of wetland soil may be caused by increased thermogenic methane emissions. From August to November, the atmospheric temperature decreased below 0 °C and the methane absorption flux gradually decreased to a minimum with this decrease in temperature, returning to the methane absorption level observed for the spring season prior to the start of the next seasonal cycle.

## Discussion

**Figure 7:**
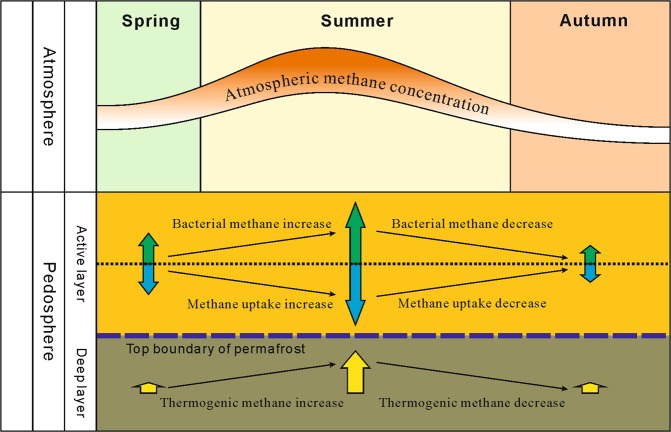
Seasonal variations of methane gas sources.

According to the above results, the seasonal variations of methane emissions can be summarized as shown in Fig. 7. During spring, microbes are the dominant sources of methane. As an increase in temperature promotes biological activity, the methane absorption effects of methanotrophic microbes and adsorption effects of the soil are enhanced, which results in a slow increase in the methane absorption flux of soil during spring (March to April). The combined effects of methane produced by methanogenic microbes and thermogenic methane lead to a lower methane content in low-level air (Fig. 4a).

During the transition from spring to summer, an exponential increase in the scale of microbial communities occurs along with the increase in temperature^48^. As the structure of methanogenic microbial communities is also influenced by increased plant productivity^19^, the methanogenic productivity of these microbes increases to a greater extent compared with the increase in the soil methane absorption ability. As the atmospheric temperature in the study area remains below 0 °C in early summer, thawing action in the permafrost is relatively weak, which leads to smaller methane contributions from thermogenic sources and a slow increase in the atmospheric methane concentration.

The average daily temperature, low-level atmospheric methane concentration, and wetland methane emissions reached a maximum during the summer months of June to August. However, previous research has shown that with an increase in temperature, to a certain extent, the upper limit of the environmental carrying capacity of methanogenic microbial communities is reached, which results in an extremely slow increase in methane concentration^38^. Therefore, the increase in atmospheric temperature does not lead to a rapid increase in wetland methane emissions. In the present study, thermogenic methane emission sites were identified in wetland soil during August through methane flux monitoring and carbon isotope analysis of the soil free gas. From this finding, we deduce that the methane emission bursts from wetlands near the end of the growing season may be attributed to three effects: (1) the adsorbed methane content in wetland soils approaches the saturation point, leading to weaker absorption effects in the soil; (2) as the temperature increases, slow increases occur in the emission of bacterially-derived methane; and (3) the depth of thawed permafrost increases, thereby enhancing the effusion of methane from subterranean gas hydrates.

From late summer to autumn and winter, decreases occur in microbial activity, the scale of microbial communities, and the absorption effects of the active layer towards methane as the temperature decreases. This leads to a substantial reduction in the amount of methane produced by methanogens. In addition, as the average daily temperature falls below 0 °C, freezing occurs in the permafrost, which leads to the decreased effusion of methane from subterranean gas hydrates. During autumn and winter, the heavy hydrocarbon content in the surface soil-adsorbed gas increases and methane from subterranean gas hydrates may seep through pathways such as drainage systems or faults, or migrate to the surface through slow diffusion effects, becoming the dominant source of wetland methane within this period.

## Conclusions

This study focused on systematic soil methane cycle geochemical monitoring in a typical gas hydrate region in the Qinghai-Tibet Plateau. The measurements presented in this work provide evidence that the effusion of natural gas hydrate underground is a methane source of wetland soil in permafrost regions. The significantly correlation between methane and heavy hydrocarbon content shows the same source of abiotic methane. The heavy value of carbon isotope content of methane in near surface gas indicates that the themogenic hydrocarbon in gas hydrate has been discharged to the surface. The δ^13^C-methane (–47.96‰ to –42.22‰ PDB) in free gas at the methane emission sites also confirmed the existence of themogenic hydrocarbon effusion. Hence, as shown in previous studies, the absorption of methane in soil active layers is an important factor affecting soil methane emissions.

Nonetheless, the process of methane emission from natural gas hydrates in the Qinghai-Tibet plateau is still not fully understood. With this in mind, the results of this research provide evidence of the effusion of thermogenically-derived methane from natural gas hydrates. These methane emissions may continue to increase as the climate warms and with further development of gas hydrate resources. Research on gas hydrate methane emissions will facilitate the understanding of carbon cycling and may allow for exploration of the mechanisms of soil methane emissions in the permafrost regions of the Qinghai-Tibet Plateau.

## Methods

### Sample collection and analysis

**Figure 8:**
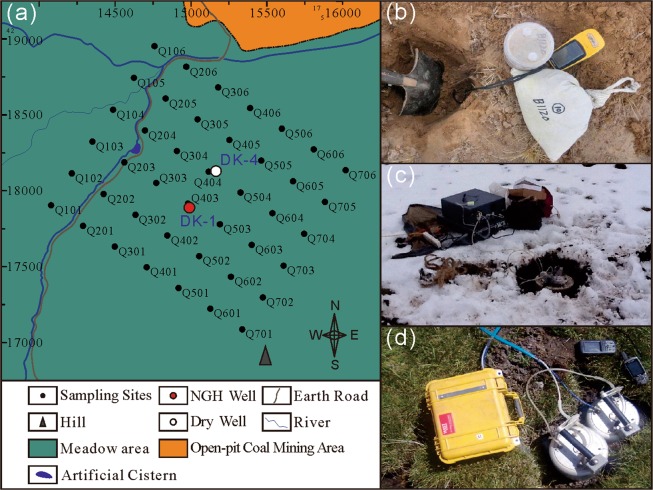
The sample collection method, (**a**) the map of sample collection sites, (**b**) carbon isotope testing, (**c**) soil sampling, and (**d**) methane flux monitoring. Sampling method photos taken by Shunyao Zhang in the study area.

As a first step in this study, a data collection zone with an area of 3 km^2^ was established. By applying a grid to the data collection zone, a total of 42 (7 × 6) monitoring sites were set up with a grid interval of 250 m (Fig. 8a). Low-level air samples from the land surface and near-surface soil samples were collected in May, August, and October 2016 and analyzed for methane content, gas chromatography (GC) of soil-adsorbed hydrocarbons, and carbon isotope content of methane. Soil-methane fluxes from the upper active layer (20–30 cm) at each of the monitoring sites were recorded in March 4–15, April 12–22, June 19–25, August 15–25, November 13–17 and December 3–7 2016.

### Methane content in low-level air and near-surface soil physically adsorbed gas

Near-surface low-level air samples were collected using the water displacement method. A 400-ml bottle filled with water was placed on the ground surface and air was collected in it via water displacement. Subsequently, the bottle was left to stand for at least 30 min to facilitate adequate exchange between the air within the bottle and low-level air at the ground surface before the bottle was sealed onsite.

Secured by the soil van der Waals force, the adsorption-desorption processes were less affected by temperature. The gas samples were collected from the clay or silt layers of the Quaternary coverage area (Fig. 2b) at a depth of 20–30 cm. After removing gravel and grass roots, each sample was placed in a bottle pre-filled with 200 ml of saturated brine. When the liquid level of the saturated brine reached the 400-ml graduation mark, the bottle was sealed, and placed in an inverted position. More details about the physically adsorbed gas sampling were described by Sun *et al*.^17^.

The methane content was measured using gas chromatography (GC) (Agilent 7890 A, USA). Before sample analysis, 20 mL ± 0.1 uL of standard gas was accurately drawn with a microsyringe and used for equipment calibration to ensure a relative error of less than 3%. After calibration, the six-port valve was heated to 100 °C ± 5 °C, the pre-column was frozen with a liquid nitrogen cold trap, and a carrier gas flow rate of 25 mL/min was used for enrichment via the six-port valve. Then, 50 mL of the gas sample were drawn using a syringe and injected into the injection port. After air separation, the six-port valve was switched to the analysis position and the GC program was initialized for quantitative calculations and plotting of the GC spectra.

### Carbon isotope content of methane in near-surface soil free gas

The stable carbon isotope content of methane in near-surface soil free gas samples was measured using a G2132-i Isotope Analyzer (Picarro, USA) (Fig. 2c), which utilizes cavity ring down spectroscopy (CRDS) and has a measurement accuracy of less than 0.8‰. The analyzer was equipped with a cylindrical stainless-steel chamber with a cross-sectional area of 0.19 m^2^, an internal volume of 19.2 L, and 1/4-in (6.35 mm) adaptor sleeves for the chamber inlet and outlet.

At each sampling site, a hole with a depth of 20–30 cm was excavated, and then, the chamber was embedded immediately and sealed with soil. The chamber was connected to the instrument, and the system was allowed to warm up for 60 min after a cold boot. After adjustments were made to the instrument, readings were collected under a high-accuracy mode at intervals of 5 min for a total duration of 60 min. The measurements were then saved and recorded on a storage card.

### Monitoring of near-surface soil-atmosphere methane flux

Near-surface soil-atmosphere methane fluxes were measured onsite (Fig. 2d) using a portable soil fluxmeter (WEST Systems WS-L1840, Italy) connected to a cylindrical stainless-steel chamber with a cross-sectional area of 0.06 m^2^ and an internal volume of 4.7 L and a 1/4-in (6.35 mm) adaptor sleeve. A module utilizing tunable diode laser absorption spectroscopy (TDLAS) was installed in the fluxmeter for continuous dynamic monitoring of methane content (ppm). The absolute atmospheric temperature (*T*_*k*_), pressure (*P*), and specific gas constant (*R*) were also recorded during the monitoring period. Using the rate of change in methane content (*F*, ppm/s), the net air inflow (*A*) and the volume (*V*) of the chamber, the surface-atmosphere methane flux (*M*, mol·m^–2^·h^–1^) was calculated using the following equation (measurement accuracy: 3% of reading, repeatability: 1.5%, methane flux measurement range: 0.5–150.0 mol·m^–2^d^–1^):

$${\rm{M}}=F\cdot \frac{86400\cdot P}{{10}^{6}\cdot R\cdot {T}_{k}}\cdot \frac{V}{A}$$

In previous studies, the carbon flux was measured between 8:00 to 10:00^49^ and 13:00 to 17:00 local standard time^50^. Since it usually rains/snows in the study area, the methane flux was monitored between 8:00 and 10:00 local standard time. At each monitoring site the air inlet was located in a pre-excavated hole with a depth of 20–40 cm. After sealing the chamber in the hole, the chamber and the instrument were connected and the system was allowed to warm up for 20 min after a cold boot. When the correlation coefficient (R) was greater than 0.8, the instrument readings were collected, saved, and recorded on a storage card.

### Data processing and analysis

All experimental data were considered for analysis; these data were collated using Microsoft Excel 2013 and differences among the seasonal data were compared using their mean and median values. Boxplots exhibiting the distribution characteristics of the data were plotted using Golden Software Grapher V12, with box boundaries being the upper and lower quartiles, and data outside the 1.5 times the interquartile range were regarded as outliers. The calculation of correlation coefficients, correlation analysis, and plotting of isotope-dryness coefficient scatter plots were all performed using Microsoft Excel 2013.

## Data Availability

The datasets analyzed during the current study are available from the corresponding author on reasonable request.
